# Identification of *LIFR, PIK3R1*, and *MMP12* as Novel Prognostic Signatures in Gallbladder Cancer Using Network-Based Module Analysis

**DOI:** 10.3389/fonc.2019.00325

**Published:** 2019-05-01

**Authors:** Xinyi Zhao, Mengxiang Xu, Zhen Cai, Wenji Yuan, Wenyan Cui, Ming D. Li

**Affiliations:** ^1^State Key Laboratory for Diagnosis and Treatment of Infectious Diseases, Collaborative Innovation Center for Diagnosis and Treatment of Infectious Diseases, The First Affiliated Hospital, Zhejiang University School of Medicine, Hangzhou, China; ^2^Research Center for Air Pollution and Health, Zhejiang University, Hangzhou, China; ^3^Institute of Neuroimmune Pharmacology, Seton Hall University, South Orange, NJ, United States

**Keywords:** functional interaction network, gallbladder cancer, module biomarker, *LIFR*, *PIK3R1*, *MMP12*

## Abstract

**Background:** Gallbladder cancer (GBC) is a rare and aggressive malignancy of the biliary tract with a dismal survival rate. Effective biomarkers and therapeutic targets are urgently needed.

**Methods:** We analyzed gene expression profiles of GBC to identify differentially expressed genes (DEGs) and then used these DEGs to identify functional module biomarkers based on protein functional interaction (FI) networks. We further evaluated the module-gene protein expression and clinical significance with immunohistochemistry staining (IHC) in a tissue microarray (TMA) from 80 GBC samples.

**Results:** Five functional modules were identified. Module 0 included classical cancer signaling pathways, such as Ras and PI3K-Akt; and modules 1–4 included genes associated with muscle cells, fibrinogen, extracellular matrix, and integrins, respectively. We validated the expression of *LIFR, PIK3R1*, and *MMP12*, which were hubs or functional nodes in modules. Compared with paired peritumoural tissues, we found that the expression of *LIFR* (*P* = 0.002) and *PIK3R1* (*P* = 0.046) proteins were significantly downregulated, and *MMP12* (*P* = 0.006) was significantly upregulated. Further prognostic analysis showed that patients with low expression of *LIFR* had shorter overall survival than those with high expression (log-rank test *P* = 0.028), the same trend as for *PIK3R1* (*P* = 0.053) and *MMP12* (*P* = 0.006). Multivariate analysis indicated that expression of *MMP12* protein (hazard ratio [HR] = 0.429; 95% confidence interval [CI] 0.198, 0.930; *P* = 0.032) was one of the significant independent prognostic factors for overall survival.

**Conclusions:** We found a highly reliable FI network, which revealed *LIFR, PIK3R1*, and *MMP12* as novel prognostic biomarker candidates for GBC. These findings could accelerate biomarker discovery and therapeutic development in this cancer.

## Introduction

Gallbladder cancer (GBC), the sixth most common gastrointestinal cancer, is an uncommon but challenging disease. Its incidence has recently increased highly worldwide (1). The risk factors for GBC include sex, aging, obesity, chronic cholecystitis, and cholelithiasis (2, 3). Because of the lack of an effective early diagnostic method, the disease often is not diagnosed until it has reached an advanced stage, when the prognosis generally is dismal, with a median survival of <1 year (3). Despite multiple studies on methods of molecular diagnosis and prediction of clinical outcomes (2), progress has been limited. More precise markers with better sensitivity and specificity are greatly needed for diagnosis and development of novel therapeutic strategies for GBC.

Recently, various network-based analytic approaches have been employed to search for signatures in microarray gene expression datasets that are related to clinical outcomes for several types of cancer (4–7). For example, Wu et al. used modules derived from a functional interaction (FI) network to identify a 75-gene signature associated with patient survival in ovarian cancer (8). Another study has indicated that multi-gene signatures are more effective for prediction than are single gene expression values (9).

To acquire further insights into the molecular mechanisms of GBC, we utilized gene expression profiles of five such cancers and five adjacent non-cancerous tissues using the Gene Expression Omnibus (GEO) database and analyzed them by constructing a protein FI network. We identified sets of genes that were either significantly downregulated or upregulated as functional module biomarkers for GBC. Finally, *LIFR, PIK3R1*, and *MMP12* were confirmed as hubs or functional nodes in modules in 80 GBC patients using immunohistochemical (IHC) technique. The relations between expression of the three proteins and patient clinical characteristics and post-operative follow-up was revealed by statistical analysis.

## Materials and Methods

### Microarray Data

The gene expression profiles of five GBCs and five matched adjacent non-cancerous tissues were downloaded from GEO with accession number GSE62335 (10). Gene expression was analyzed using an Affymetrix Human Gene 2.0 ST array (Affymetrix Inc., Santa Clara, CA, USA). The raw CEL data were downloaded, and Robust Multi-Array Average (RMA) methods and the “Oligo” package from BioConductor (http://www.bioconductor.org) were used to normalize the data and annotate the probe information.

### Initial Data Processing and Identification of DEGs

Normalized signal intensity data were imported into BRB-ArrayTools (v. 4.5; http://linus.nci.nih.gov/BRB-ArrayTools.html) for initial processing. We excluded those genes with more than 50% of data missed. We identified differentially expressed genes (DEGs) using a paired *t*-test with a random variance model, which is an improvement over the standard separate *t*-test when it is applied in small-sample microarray experiments (11). An adjusted-*P* value of 0.05 was considered nominally significant for each tested gene. Only genes with a fold change ≥2 and an adjusted-*P* value of < 0.05 were selected as DEGs.

### Construction of an FI Network

A total of 217,249 pairs of FIs were downloaded from Reactome (v. 2014; http://www.reactome.org) (12). These pairwise relations are derived from datasets of protein–protein interactions from BioGrid (13), the Database of Interacting Proteins (14), the Human Protein Reference Database (15), I2D (16), IntACT (17), and MINT (18), as well as from gene co-expression data derived from multiple high-throughput techniques, including yeast two-hybrid assays, mass spectrometry pull-down experiments, and DNA microarrays (19). The above interaction information was imported into Cytoscape software (v. 3.2.1; http://www.cytoscape.org) to construct the FI network (20).

### Pathway and Gene Ontology (GO) Term Enrichment Analysis for the FI Network

The ReactomeFIViz app was used in Cytoscape for pathway and GO enrichment analysis (21). The sources of pathway annotations included CellMap (http://www.pathwaycommons.org/pc/dbSnapshot.do?snapshot_id=8), Reactome (12), Kyoto Encyclopedia of Genes and Genomes (KEGG) (22), Panther Pathways (23), NCI–PID (24), and BioCarta (http://www.biocarta.com/genes/index.asp). A false discovery rate (FDR) of <0.05 was accepted as the cut-off criterion.

### Identification of a Network-Based Functional Module

The Microarray Data Analysis tool from ReactomeFIViz was used for network-based functional analysis (21). The gene expression data were first loaded into this bioinformatics tool. Second, correlations among the genes were calculated in the whole FIs network. Finally, we applied the Markov cluster algorithm (MCL) to generate modules by selecting the threshold of a module size or an average correlation value. To control the size of network modules generated from MCL clustering, we used 5.0 as the inflation coefficient. For GBC data analysis, we chose MCL modules of size three or greater and an average Pearson correlation coefficient (PCC) ≥ 0.25 and chose an absolute value for edge weights. Nodes in different network modules are shown in different colors. Pathway or GO term enrichment analysis was used for each individual network module. We selected a size ≥3 as a cutoff to filter out network modules and chose an FDR <0.05 as a cutoff for viewing enriched pathways or GO terms.

### Patient Samples

For tissue microarray (TMA) detection, 80 human GBC specimens, 20 of which had matched non-tumor tissues, were collected between 2007 and 2012 at Taizhou People's Hospital of China. All tissues were stored at the Biobank Center of National Engineering Center for Biochips at Shanghai in China. None of these patients received any preoperative anticancer therapy or post-operative adjuvant chemotherapy. The clinical pathologic features of patients are given in Table S1. The tumor differentiation grade and clinical stages were classified according to the American Joint Committee on Cancer (AJCC) TNM Classification (7th edition). We calculated the follow-up time from the date of surgery to the date of death or last visit. The use of human tissue samples and clinical data were approved by the Ethical Committees of the National Engineering Center for Biochips at Shanghai and Taizhou People's Hospitals. All donors had provided written informed consent for donating their tissues to research-related activities.

### TMA Construction

The GBC tissue microarrays were constructed using tissue cores from Formalin-fixed, paraffin-embedded specimens. Representative cancer tissue regions and para-cancerous non-malignant or non-premalignant lesions gallbladder specimens were selected from each tissue block by licensed pathologists, and a single 1.5-mm core was taken from every donor block. Microarray blocks were constructed using an automated tissue arrayer (Beecher Instruments, Sun Prairie, WI, USA). Five-micrometer sections were dissected from the array blocks. Sections were stained with hematoxylin and eosin (H&E) to confirm the presence of tumor in each core.

### IHC Staining

The IHC staining was conducted as described previously (25). To summarize, tissue sections were incubated at 4°C overnight with rabbit anti-LIFR diluted 1:1,500 (Abcam, Cambridge, UK), rabbit anti-PI3 kinase p85 alpha diluted 1:600 (Abcam), or rabbit anti-MMP12 diluted 1:100 (Abcam). The percentage of immunostaining and the staining intensity (0 = no staining, 1 = weak, 2 = moderate, and 3 = strong staining) were recorded. An H-score was calculated as follows: H-score = (% cells of 1 intensity × 1) + (% cells of 2 intensity × 2) + (% cells of 3 intensity × 3) (26). The maximum H-score would be 300, corresponding to 100% of cells with strong intensity. In statistical analysis, patient characteristics were compared according to the H-score when dichotomizing by the median value in 80 GBC patients. The IHC H-scores were determined independently by two pathologists, who were blinded to the patients' clinical data.

### Statistical Analysis

SPSS 21.0 (SPSS Inc.; Chicago, IL, USA) and GraphPad Prism 6 (San Diego, CA USA) software were used for statistical analysis and graphic representations. The χ^2^ test was used to analyze the relations between protein expression and clinicopathologic features in GBC patients. Survival curves were evaluated using the Kaplan-Meier method, and differences between survival curves were tested by the log-rank test. Cox proportional hazards regression was used to examine univariate and multivariate analyses. The Forward Likelihood Ratio method was used to select independent variables in multivariate analysis.

### Expression of *LIFR, PIK3R1*, and *MMP12* From the Cancer Genome Atlas (TCGA) and Gene Expression Omnibus (GEO) Datasets

*LIFR, PIK3R1*, and *MMP12* gene expression pattern in different gastrointestinal cancers were conducted on the TCGA dataset (27). We compared the three gene expression between cancer tissues and their adjacent non-cancerous tissues in four types of cancers, which included cholangio carcinoma, liver hepatocellular carcinoma, pancreatic adenocarcinoma, and stomach adenocarcinoma.

In addition, we found another public GBC dataset and used it for validation of our findings. This dataset was downloaded from the NCBI GEO database with the accession number of GSE76633 and GPL18180. For details on this dataset, please referred to the original report (28). Unpaired Student's t-test was used for this part of analysis, with a *p*-value < 0.05 being considered as significant.

## Results

### Identification of DEGs

The gene expression profile of GSE62335 was downloaded from the GEO database, and a random-variance mode method was used to identify DEGs in GBC compared with non-cancerous controls using a paired *t*-test with a random variance model (11). With the criteria of a fold change ≥ 2 and adjusted-*P* < 0.05, 198 genes were identified as DEGs (Table S2). Of these, 66 (33.3%) were upregulated, and the remaining 132 (66.7%) were downregulated (Table S2).

### Construction of a GBC-Related FI Network

**Figure 1:**
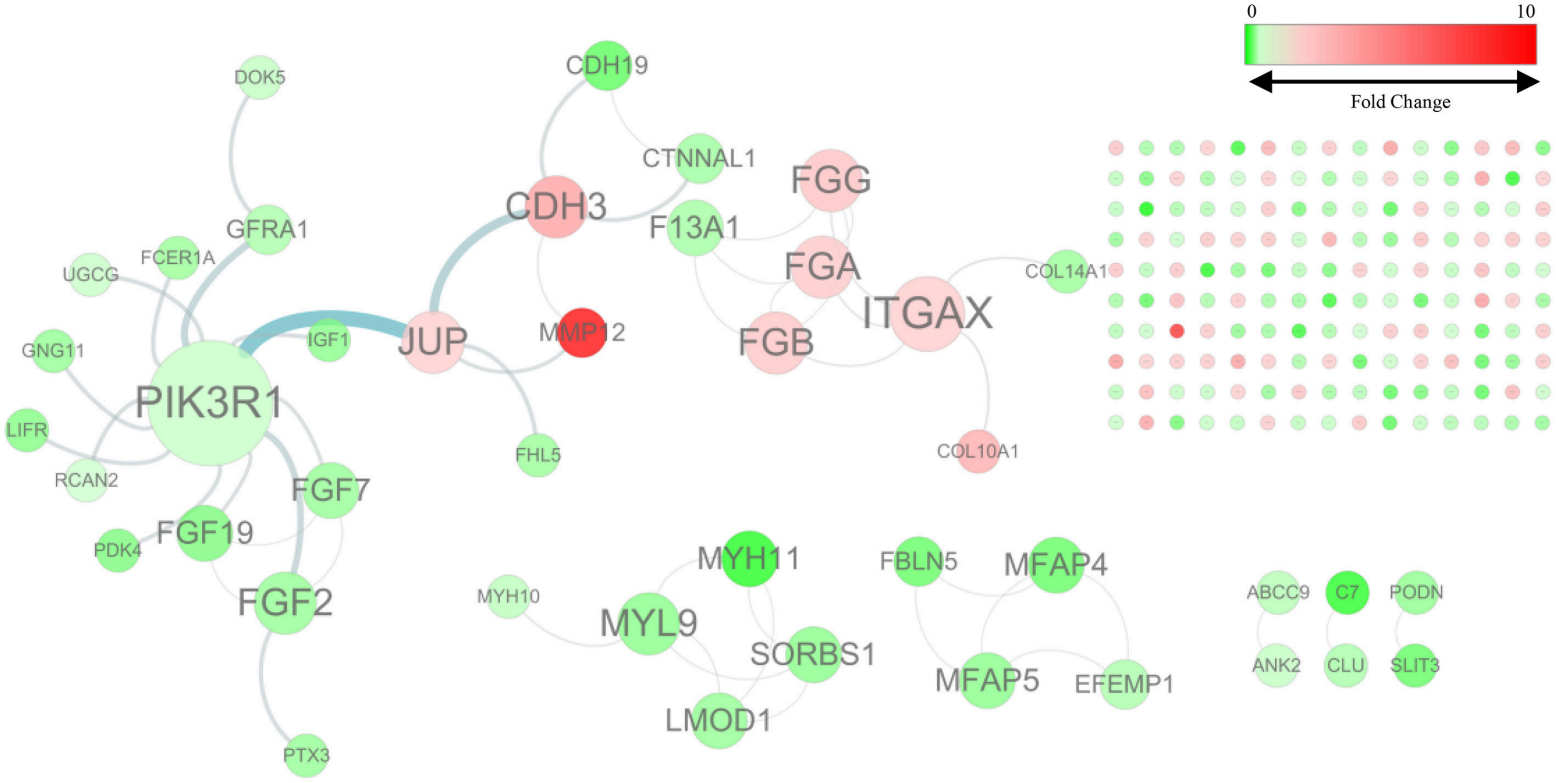
The functional interaction (FI) network constructed using GBC-related differentially expressed genes. Edges are based on FI annotation. Node sizes represent degree value of the FI network, ranging from 0 to 12. Node colors define fold changes in GBC-related DEGs, ranging from red for high expression to green for low expression, relative to non-cancerous samples.

By mapping the GBC-related DEGs to the FI data, we constructed a GBC-related FI network. The network comprises 192 nodes: 150 isolated and 42 in seven clusters, with the largest cluster containing 20 nodes (Figure 1). These clustered nodes are connected via 50 FIs, which correspond to an effective mean degree of 1.2. “Degree” refers to the number of nearest neighbors of a node and effective mean degree to the average degree of all nodes other than the isolated ones. Nodes whose degrees scored ≥4 were selected as hub nodes. Under these criteria, we found that *PIK3R1, ITGAX, CDH3, FGA, FGB, FGF2, FGG, JUP*, and *MYL9* were hub nodes in the FI network, suggesting that these genes play important roles in the initiation of GBC.

### Analysis of FI Network Functions

To classify these 192 significant genes functionally, we used ReactomeFIViz to identify significant enrichment of these genes in six pathways (see Table S3). The most significant pathway was extracellular matrix organization. The GO result showed that the most significant functional groups consisted of genes involved in extracellular matrix organization (biological process), extracellular vesicular exosomes (cellular component), and calcium-ion binding (molecular function) (see Tables S4–S6).

### Construction of Network-Based Functional Modules

**Figure 2:**
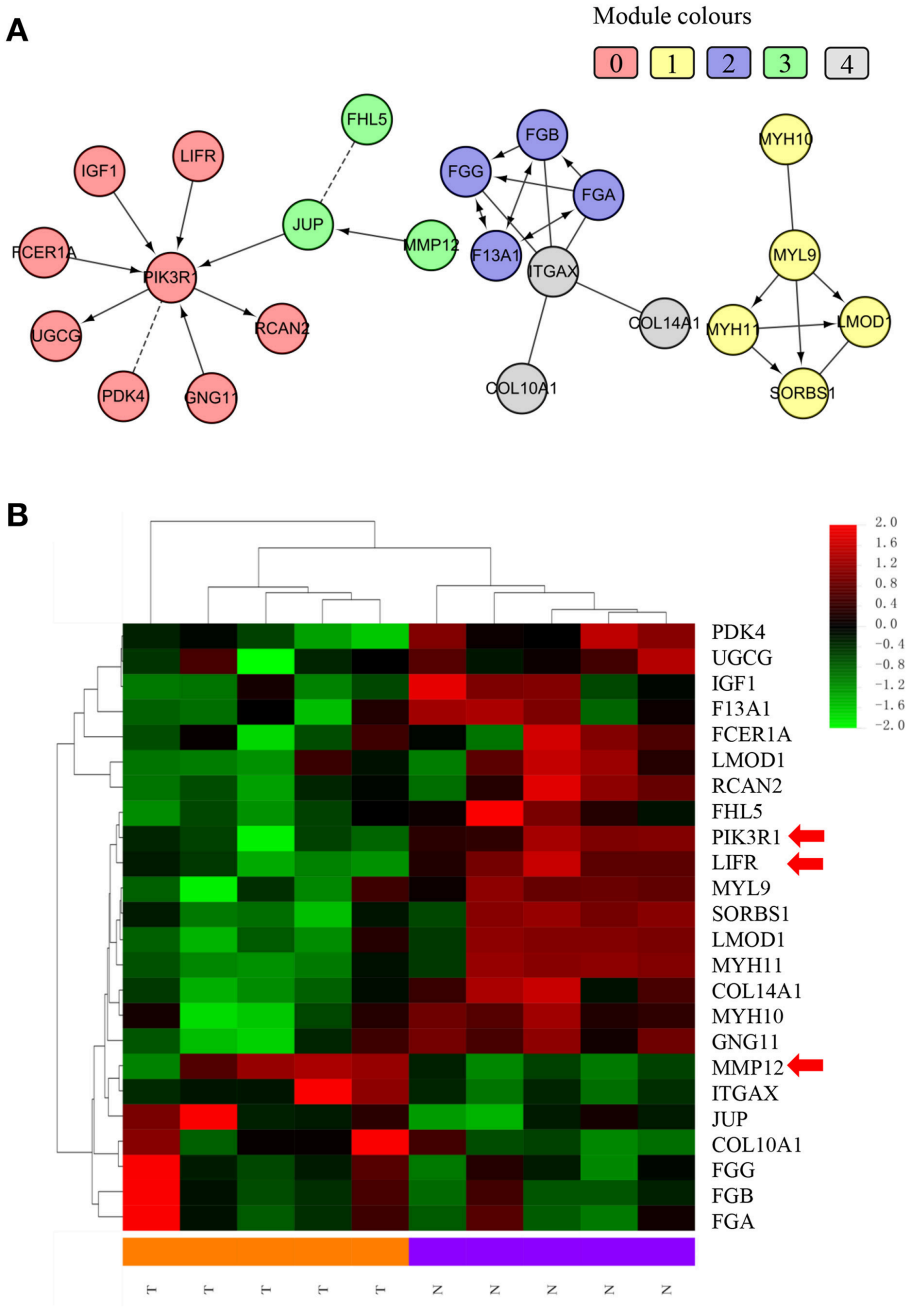
Functional interaction sub-network constructed using module genes. **(A)** The five generated network modules comprise 23 genes, which are shown in different colors in different network modules. **(B)** Heat map of the 23 genes differentiating GBC samples from non-cancerous samples.

We chose the MCL as the network-clustering algorithm to take advantage of edge weights. We weighted each interaction edge according to the absolute value of the PCC of the extent of expression of the two genes connected by the edge. For GBC microarray data analysis, we chose MCL modules ≥ 3 and average PCCs ≥ 0.25. In the discovery set, five modules ranging in size from three to eight genes passed the filters (Table 1). An FI sub-network was constructed using 23 genes comprising five modules (Figure 2A). Using hierarchical clustering based on the extent of gene expression (Figure 2B), we found that the 23 genes in the modules could be used to differentiate GBC from non-cancerous samples.

**Table 1:** Genes in modules in FI network.

**Module**	**Number of genes**	**Average correlation**	**Gene set**
0	8	0.4831	*FCER1A, GNG11, IGF1, LIFR, PDK4, PIK3R1, RCAN2, UGCG*
1	5	0.7697	*LMOD1, MYH10, MYH11, MYL9, SORBS1*
2	4	0.5426	*F13A1, FGA, FGB, FGG*
3	3	0.6762	*FHL5, JUP, MMP12*
4	3	0.3151	*COL10A1, COL14A1, ITGAX*

### Biological Role of Functional Modules

To understand how the 23 genes of the five modules might be related to the molecular mechanisms of GBC, we performed a functional enrichment analysis on these modules based on pathway annotation (Table S7). The enriched pathways of Module 0 were related mainly to classical cancer pathways, such as Ras, PI3K-Akt, or mTOR signaling in KEGG and integrins in angiogenesis in NCI-PID. Modules 1 and 2 were related to muscle-specific genes and fibrinogen, respectively. The enriched pathway of Module 3 was that of Alzheimer disease–presenilin in NCI-PID, within which *MMP12* was related to extracellular matrix also. Extracellular matrix organization in the reactome and the integrin signaling pathway in Panther were enriched in Module 4. An enrichment analysis of GO annotations (biological process, cellular component, molecular function) indicated that the GO biological functions of the modules were consistent mainly with pathway annotations (see Tables S8–S10).

### Validation of Differential Expression of *LIFR, PIK3R1*, and *MMP12* as Candidate Module-Biomarkers in GBC

According to the function of the modules and the degree value of the nodes in the GBC-related FI network, we chose *PIK3R1* (degree = 8) as a functional hub gene in Module 0, which was the most related to classical cancer pathways (29–31), to validate expression in GBC. Moreover, *LIFR* was selected as the gene most relevant to *PIK3R1* on the basis of the FI data of network and cluster correlation (Figure 2). Finally, the most upregulated gene, *MMP12*, was validated as a candidate indicator of the extracellular matrix pathway that was the most significantly enriched in the functional network, having an indirect effect on *PIK3R1* through *JUP*.

**Figure 3:**
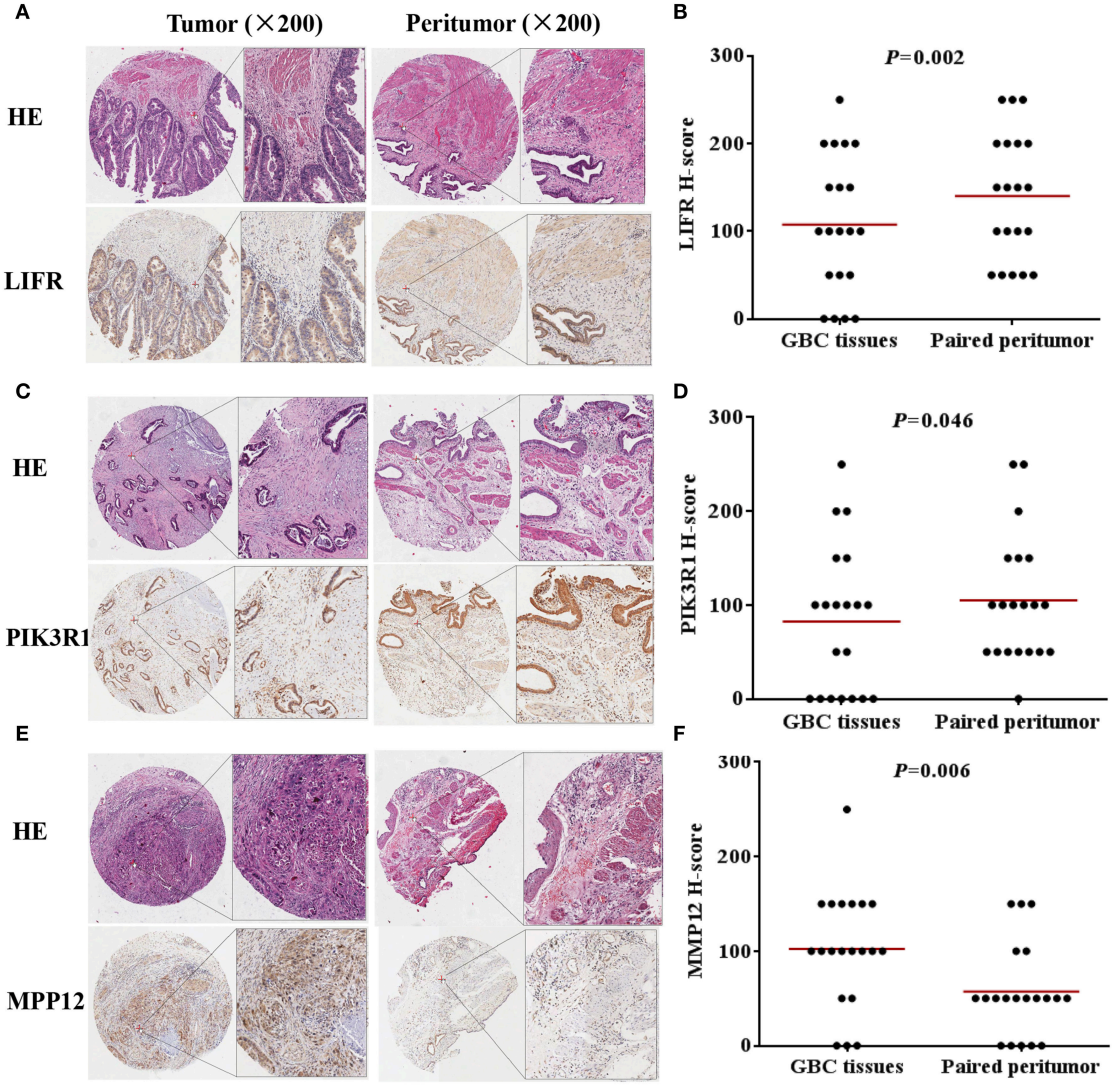
*LIFR, PIK3R1*, and *MMP12* expression in gallbladder cancer tissue. Immunostaining of *LIFR* **(A)** expression in a representative tumor tissue and matched non-tumor tissue; original magnification ×200. **(C,E)** are representative images of *PIK3R1* and *MMP12*, respectively. The differential protein expression of *LIFR* **(B)**, *PIK3R1* **(D)**, and *MMP12* **(F)** are shown in GBC tissues and matched non-tumor tissues of 20 patients as indicated. The median extent of expression of each protein is indicated by the horizontal line in the scatterplot figure.

We used IHC methods to determine LIFR, PIK3R1, and MMP12 protein expression in 20 GBC tumor tissues and patient-matched adjacent peritumor tissues. *LIFR* protein expression was significantly downregulated in 65% of the cases of GBC (13/20) (Figure 3B; *P* = 0.002). Similarly, *PIK3R1* protein expression was significantly downregulated in 60% of cases (12/20) (Figure 3D; *P* = 0.046). However, *MMP12* was significantly upregulated in 60% compared with paired peritumoral tissues (Figure 3F; *P* = 0.006). Representative images show that *LIFR* (Figure 3A) and *PIK3R1* (Figure 3C) expression was located mainly in the plasma membrane or cytosol, yet *MMP12* (Figure 3E) was expressed mainly in the extracellular region of GBC tissue. Furthermore, the expression data of these proteins were consistent with the mRNA expression trends of *LIFR, PIK3R1*, and *MMP12*, as determined by the microarray data (Figure 2B).

### Relation Between Expression of the Proteins and Clinical Features in GBC

To determine the clinical significance of *LIFR, PIK3R1*, and *MMP12* expression in GBC, the χ^2^ test was used to assess the associations between expression of the three proteins and clinicopathologic features (patient age; sex; extent of histologic differentiation; tumor size; T, N, and M classification; and clinical stage). We found that *LIFR* expression in GBC tissues was closely associated with tumor size (*P* = 0.035), as was *PIK3R1* (*P* = 0.040). However, no significant associations were detected between *MMP12* expression and any clinicopathologic feature such as histologic differentiation; tumor size; T, N, or M classification; or clinical stage (Table 2).

**Table 2:** Relation between expression of LIFR, PIK3R1, and MMP12 proteins and clinical characteristics of GBC.

**Factor**	**Patients**		**LIFR expression**			**PIK3R1 expression**			***MMP12*** **expression**		
	**No**.	**%**	**Low**	**High**	***P*-value**	**Low**	**High**	***P*-value**	**Low**	**High**	***P*-value**
Age (years)					0.817			0.653			0.057
≤ 65	44	55	15	22		16	21		8	29	
>65	35	43.75	15	27		21	21		18	24	
Sex					0.320			1.000			0.195
Female	56	70	24	32		26	30		21	35	
Male	24	30	7	17		11	13		5	19	
Histologic differentiation					0.182			0.423			0.105
Well	5	6.25	0	5		1	4		0	5	
Moderately	50	62.5	21	29		25	25		20	30	
Poorly	25	31.25	10	15		11	14		6	19	
Tumor size (cm)					**0.035**			**0.040**			0.230
≤ 4.5	42	52.5	10	28		13	25		10	28	
>4.5	35	43.75	20	19		23	16		16	23	
T class					0.423			0.139			0.578
1	6	7.5	2	4		2	4		2	4	
2	23	28.75	9	14		13	10		7	16	
3	31	38.75	16	15		21	10		15	16	
4	2	2.5	0	2		0	2		1	1	
*N* class					0.480			0.875			0.593
0	46	57.5	18	28		23	23		16	30	
1	12	15	7	5		7	5		4	8	
2	2	2.5	1	1		1	1		0	2	
Distant metastasis					0.498			1.000			0.720
No	70	87.5	26	44		32	38		22	48	
Yes	10	12.5	5	5		5	5		4	6	
TNM stage					0.430			0.307			0.910
I	5	6.25	1	4		2	3		2	3	
II	13	16.25	5	8		8	5		4	9	
III	21	26.25	12	9		15	6		9	12	
IV	14	17.5	6	8		6	8		5	9	

*TNM, tumor–node– metastasis according to classification system of the American Joint Committee on Cancer (AJCC) (7th Edition). P-values <0.05 in boldface type show statistical significance*.

### Correlation Between Expression of Proteins and Prognosis

**Figure 4:**
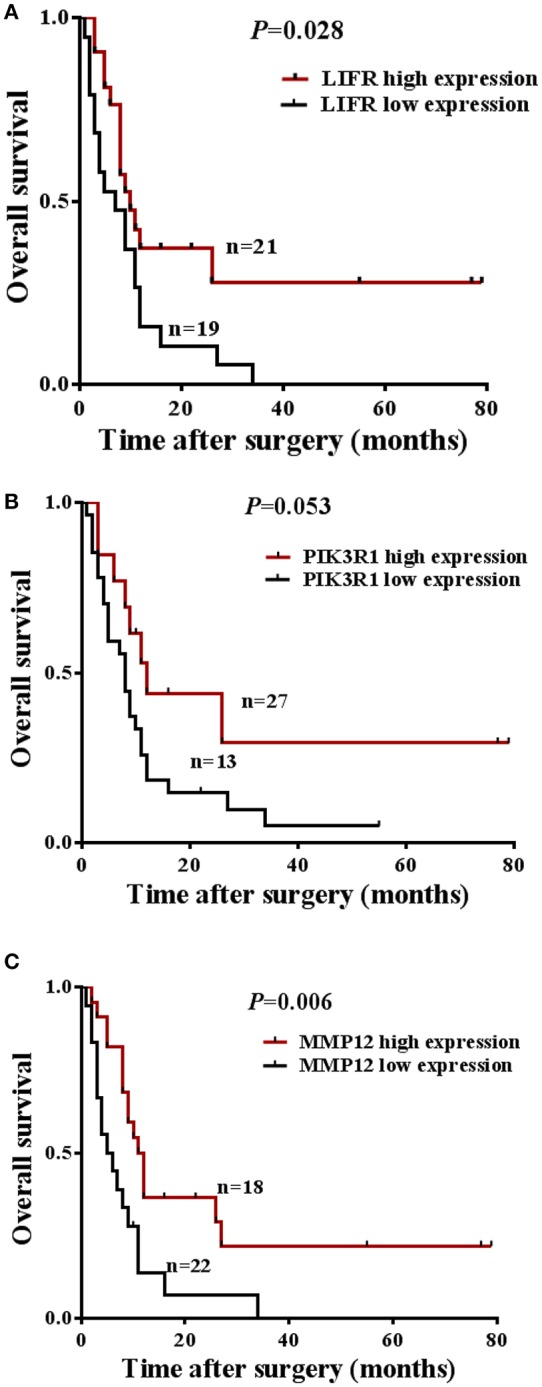
Prognostic significance of *LIFR, PIK3R1*, and *MMP12* expression for GBC patients. **(A)** Kaplan-Meier curve for the overall survival (OS) of patients with low vs. high expression of *LIFR* (median OS 7.0 vs. 10.0 months, respectively; log-rank test, *P* = 0.028). **(B)** Kaplan-Meier curve for the OS of patients with low vs. high expression of *PIK3R1* (median OS 8.0 vs. 12.0 months, respectively; log-rank test, *P* = 0.053). **(C)** Kaplan-Meier curve for the OS of patients with low vs. high expression of *MMP12* (median OS 5.5 vs. 11.5 months, respectively; log-rank test, *P* = 0.006).

The prognostic significance of *LIFR, PIK3R1*, and *MMP12* expression was evaluated with IHC values using the median value as the cutoff. The observation time was 79 months. We explored the correlation between the three proteins and clinical survival data by Kaplan-Meier analysis and the log-rank test. As shown in Figure 4A, patients with low expression of *LIFR* had poorer overall survival (OS) than those with high expression (Log-rank test, *P* = 0.028), as also was true for *PIK3R1* (Figure 4B; *P* = 0.053) and *MMP12* (Figure 4C; *P* = 0.006). The median OS of the patients with the low and high *LIFR* expression was 7.0 vs. 10.0 months, respectively. The median OSs in patients with low and high expression of *PIK3R1* and *MMP12* were 8.0 vs. 12.0 months and 5.5 vs. 11.5 months, respectively. In addition, univariate and multivariate analyses showed that *MMP12* could be useful as an independent risk factor for prognosis. Univariate Cox regression analyses showed that histologic differentiation (*P* = 0.035), T classification (*P* = 0.038), distant metastasis (*P* = 0.025), clinical TNM stage (*P* = 0.007), *LIFR* expression (*P* = 0.039), and *MMP12* expression (*P* = 0.01) were all significantly related to OS. Multivariate Cox regression analyses showed that distant metastasis (*P* = 0.038) and *MMP12* expression (*P* = 0.032) were significantly different (Table 3).

**Table 3:** Univariate and multivariate Cox regression analyses for overall survival in GBC.

**Factor**	**Univariate**		**Multivariate**	
	**HR (95% CI)**	***P*-value**	**HR (95% CI)**	***P*-value**
Age (years; ≤ 65/>65)	0.924 (0.465, 1.834)	0.820		
Sex (female/male)	1.404 (0.675, 2.920)	0.364		
Histologic differentiation (well/moderately/poorly)	2.181 (1.057, 4.502)	**0.035**		
Tumor size (cm; ≤ 4.5/>4.5)	1.498 (0.692, 3.244)	0.305		
T class (1/2/3/4)	1.728 (1.032, 2.893)	**0.038**		
N class (0/1 + 2)	2.158 (0.850, 5.479)	0.106		
Distant metastasis (no/yes)	3.209 (1.158, 8.890)	**0.025**	3.473 (1.071, 11.263)	**0.038**
TNM stage (I/II/III/IV)	1.993 (1.208, 3.288)	**0.007**		
LIFR expression (low/high)	0.481 (0.241, 0.963)	**0.039**		
PIK3R1 expression (low/high)	0.476 (0.214, 1.058)	0.068		
*MMP12* expression (low/high)	0.401 (0.199, 0.806)	**0.010**	0.429 (0.198–0.930)	**0.032**

*95% CI, 95% confidence interval; HR, hazard ratio; TNM, tumor–node–metastasis; P-values < 0.05 in boldface type show statistical significance*.

### Expression and Regulation Relation Among *LIFR, PIK3R1*, and *MMP12*

**Figure 5:**
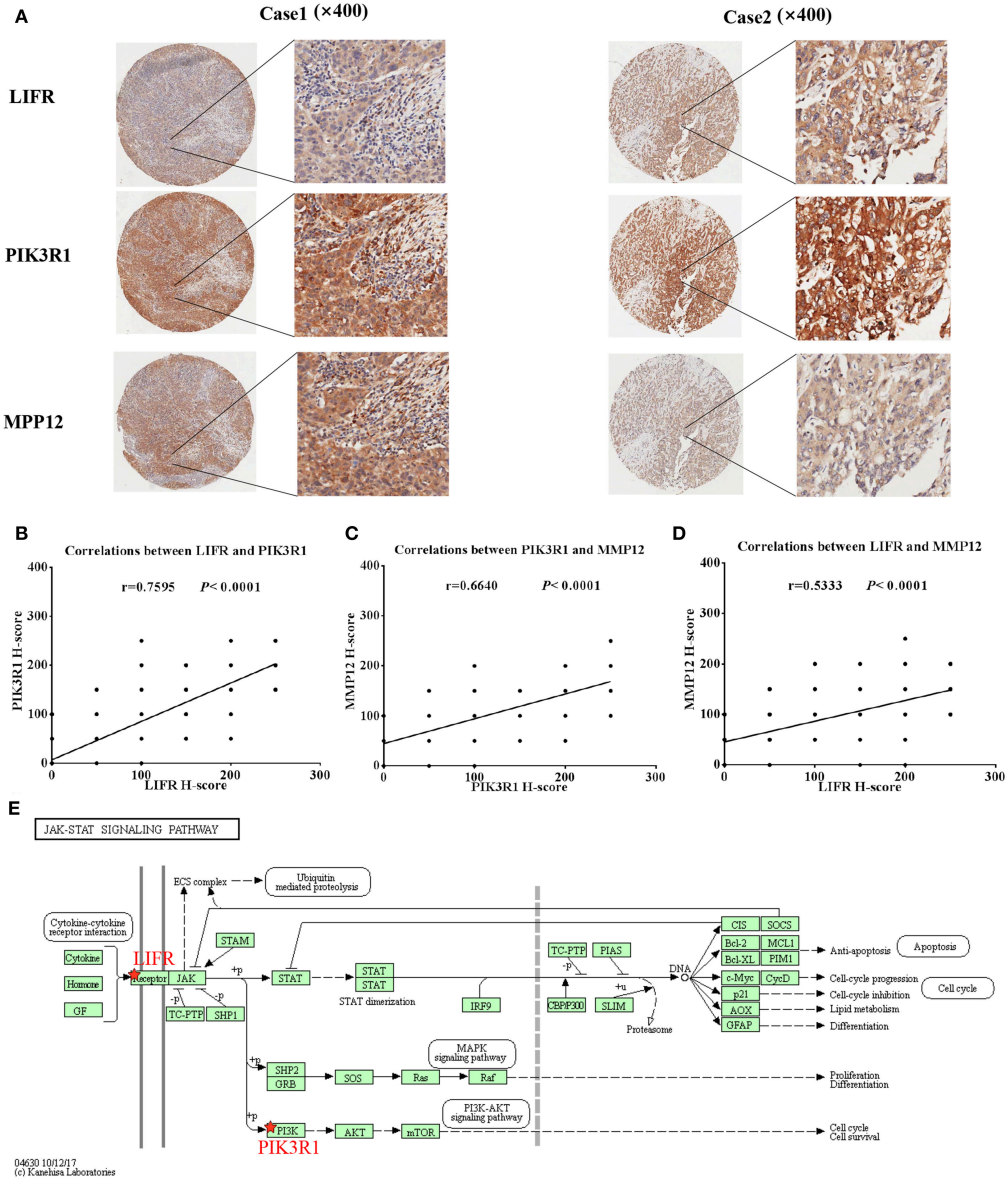
Expression and regulation relation among *LIFR, PIK3R1*, and *MMP12* in GBC patients. **(A)** *LIFR, PIK3R1*, and *MMP12* colocalized in two representative cases of GBC tissues were detected by immunostaining. Original magnification ×400. **(B)** Pearson's correlation analysis of *LIFR* expression with *PIK3R1* expression in 80 GBC patients. **(C)** and **(D)** are representative of the relation between *LIFR* with *MMP12* and relation between *PIK3R1* with *MMP12*, respectively. **(E)** Regulation relation between *LIFR* and *PIK3R1* in Jak-STAT signaling pathway of KEGG.

Because *LIFR, PIK3R1*, and *MMP12* had protein–protein interactions in the GBC-related FI network, we next determined whether the three proteins exhibited expression correlation or direct regulation. By using IHC analysis in GBC tissues, we found that *LIFR* was colocalized with *PIK3R1* in the plasma membrane or cytosol and that this colocalization was decreased in *MMP12* compared with *LIFR* or *PIK3R1* (Figure 5A). Interestingly, the expression of *LIFR* was the most positively correlated with *PIK3R1* (*r* = 0.76; *P* < 0.0001; Figure 5B) compared with correlation between *MMP12* and *LIFR* (*r* = 0.53; *P* < 0.0001; Figure 5C) or *PIK3R1* (*r* = 0.66; *P* < 0.0001; Figure 5D) in 80 GBC patients. Furthermore, we searched the *LIFR*-*PIK3R1* information of edges in the network, which was recorded in the Jak-STAT signaling pathway of KEGG (Figure 5E). These data suggested that *LIFR* directly regulates *PIK3R1* by protein–protein interactions.

### *LIFR, PIK3R1*, and *MMP12* Gene Expression Pattern in Common Gastrointestinal Cancers

**Figure 6:**
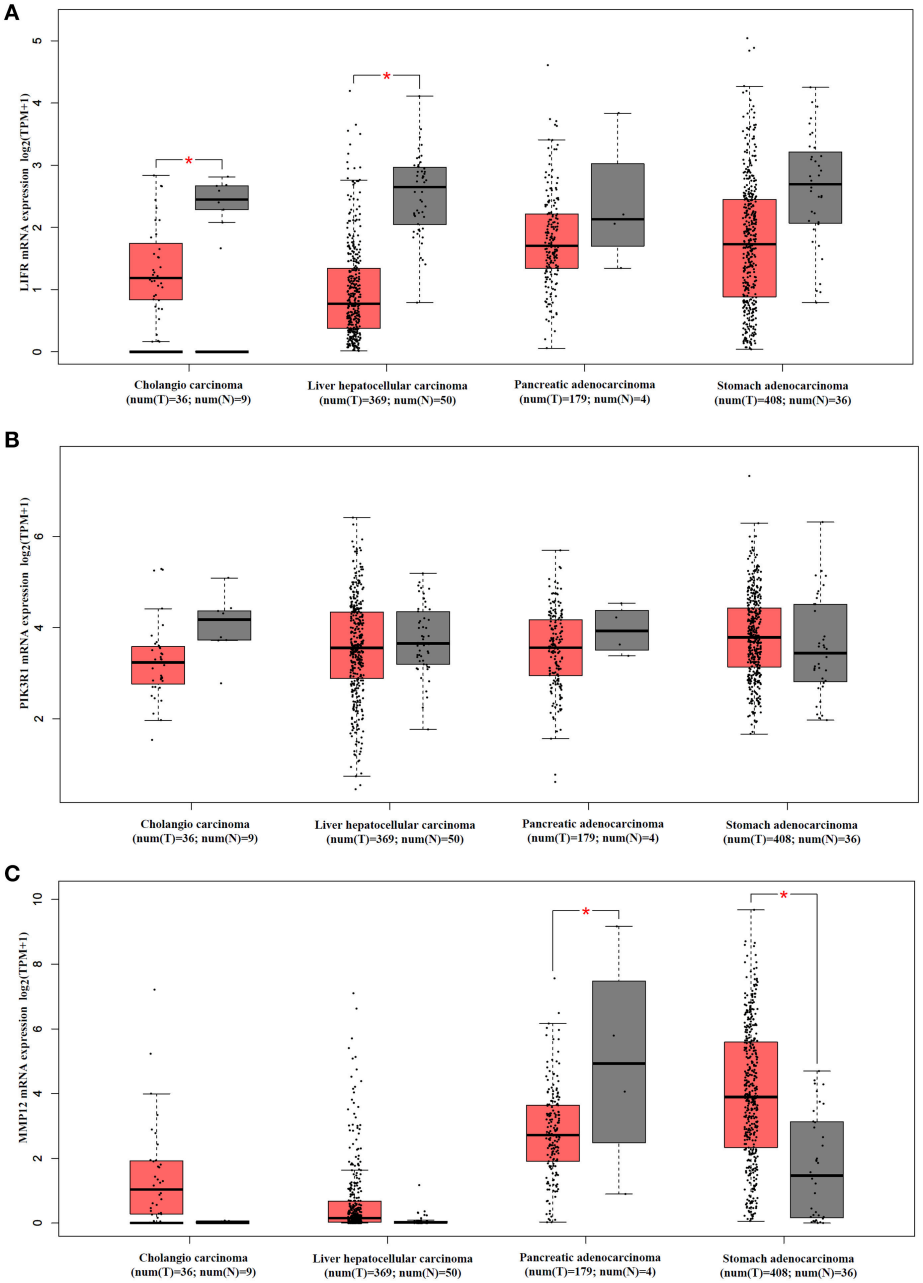
mRNA expression profile of *LIFR, PIK3R1* and *MMP12* in cholangio carcinoma, liver hepatocellular carcinoma, pancreatic adenocarcinoma and stomach adenocarcinoma. Expression profile of *LIFR* **(A)**, *PIK3R1* **(B)**, and *MMP12* **(C)** across all tumor samples and paired normal tissues. *P < 0.05.

We analyzed mRNA expression levels of *LIFR, PIK3R1* and *MMP12* in cholangio carcinoma, liver hepatocellular carcinoma, pancreatic adenocarcinoma, and stomach adenocarcinoma, with the data from the TCGA. The results showed that *LIFR* was significantly downregulated in cholangio carcinoma and liver hepatocellular carcinoma although a downregulated trend of RNA expression was detected in all four types of cancers (Figure 6A). *PIK3R1* showed no significant differences in all four types of cancers (Figure 6B), indicating that *PIK3R1* was specific to GBC. *MMP12* was significantly downregulated in pancreatic adenocarcinoma, but significantly upregulated in stomach adenocarcinoma (Figure 6C, *P* < 0.05), suggesting that the role of *MMP12* in the progression of cancer differed among different types of cancers.

### Validation of Differential Expression of *LIFR, PIK3R1*, and *MMP12* in Another GBC Dataset

**Figure 7:**
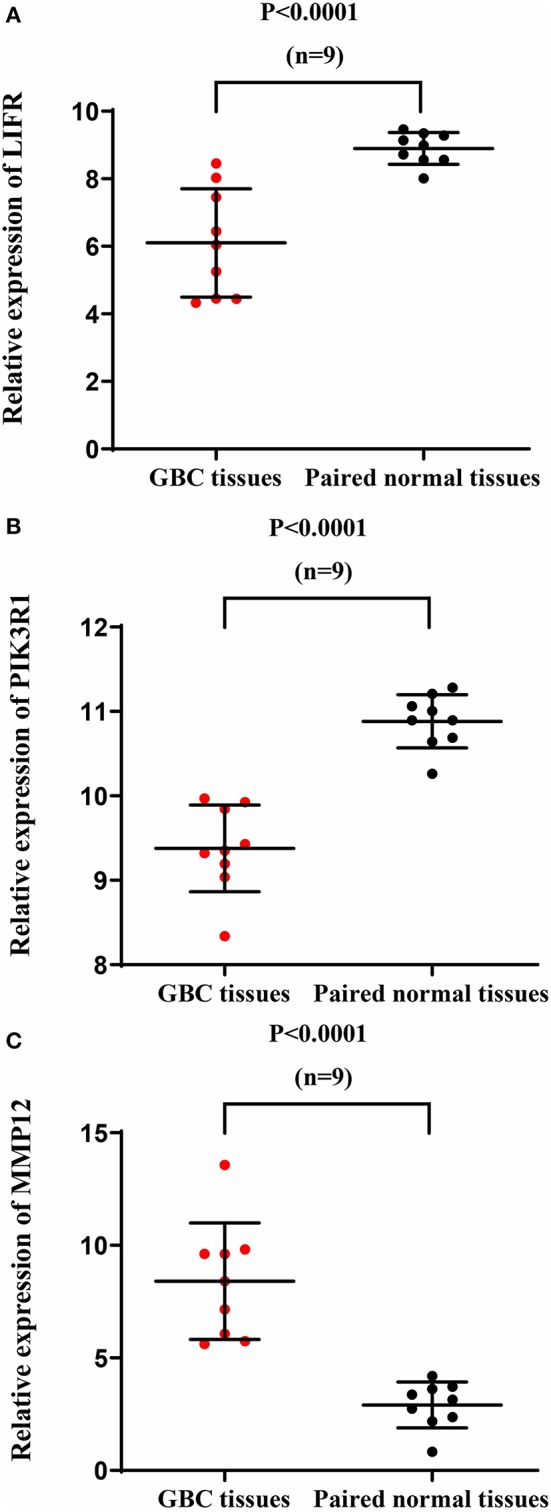
mRNA expression of *LIFR, PIK3R1*, and *MMP12* in an independent GBC dataset (GSE76633). mRNA expression profile of *LIFR* **(A)**, *PIK3R1* **(B)**, and *MMP12* **(C)** across tumor samples and paired normal tissues.

We used another GBC dataset from GSE76633 to validate our original findings. Our statistical analysis showed that the mRNA expression of *LIFR* and *PIK3R1* was significantly downregulated whereas that of *MMP12* was significantly upregulated, compared with their corresponding adjacent normal tissues (Figure 7; *P* < 0.001 for all the three genes). The expression trends at the mRNA level of each gene in this independent dataset were consistent with our detected protein expression data in our sample (Figure 3).

## Discussion

Gallbladder cancer is a highly malignant and quickly fatal disease, with a mean 5-year survival rate of only 5% (32). Its dismal prognosis is attributed to its late presentation, early lymph node metastases, frequent adjacent organ invasion, and poor response to chemotherapy. To improve the prognosis of GBC, it is important to understand the molecular mechanism relevant to gallbladder carcinogenesis and to establish appropriate molecular biomarkers enabling early diagnosis and progress monitoring.

A network-based analytic approach of microarray gene expression datasets offers a novel approach for systematically characterizing the underlying molecular etiology of malignancies and searching for gene signatures (19). Based on the microarray data, in this study, we built a highly reliable FI network to identify functional module biomarkers for GBC. According to the function of the modules and the degree value of the nodes in the network, *LIFR, PIK3R1*, and *MMP12* as novel signatures in modules are further confirmed by IHC.

The central gene is *PIK3R1* in Module 0, which module contains eight DEGs related to classical cancer pathways (*FCER1A, GNG11, IGF1, LIFR, PDK4, PIK3R1, RCAN2*, and *UGCG*). *PIK3R1* encodes the p85 regulatory subunit-α, which plays a tumor suppressor role, regulating and stabilizing the p110α catalytic subunit encoded by the oncogene *PIK3CA* (33, 34). In our study, the mRNA and protein expression of *PIK3R1* is differentially downregulated in GBC compared with non-cancerous samples (see Figures 2B, 3D). As shown in Figure 4B, it is clear that patients with low expression of *PIK3R1* have a poorer OS than those with high expression (*P* = 0.053). Moreover, *PIK3R1* is related to tumor size (*P* = 0.040) in clinical significance analysis. These data suggest that *PIK3R1* plays a tumor suppressor role in GBC. Similarly, in other cancers, *PIK3R1* downregulation has been proved to be an independent prognostic marker in breast cancers (35) and to promote propagation, migration, epithelial–mesenchymal transition, and a stem-like phenotype in renal cancer cells through the AKT/GSK3beta/CTNNB1 pathway (36). Importantly, we found *PIK3R1* showed no significant differences in other four types of cancers, suggesting that *PIK3R1* was specific to GBC among these common gastrointestinal cancers.

Another interesting gene is leukemia inhibitory factor receptor alpha (*LIFR*), which was positively correlated with *PIK3R1* in our predicted and experimental results. The mRNA and protein expression of *LIFR* is differentially downregulated in GBC (see Figures 2B, 3B). Decreased *LIFR* expression correlated with a poor prognosis (Figure 4A; *P* = 0.028) and was related to tumor size (*P* = 0.035). Consistent with our investigation, *LIFR* has been proved to be a cancer metastasis suppressor that inhibits both local invasion and metastatic colonization in a variety of tumors (37, 38). Moreover, downregulation of *LIFR* expression appeared to be common in other four gastrointestinal cancers, especially in cholangio carcinoma and liver hepatocellular carcinoma (Figure 6A). Excitingly, we find that *LIFR* was colocalized with *PIK3R1* in the plasma membrane or cytosol, and the expression of *LIFR* was significantly correlated with *PIK3R1* (*r* = 0.76; *P* < 0.0001; Figure 5B). In addition, the predicted FI information showed that a receptor complex including *LIFR* activates a PIK3 complex containing *PIK3R1* in the Jak-STAT signaling pathway of KEGG (Figure 5E). These data suggest that *LIFR* directly regulates *PIK3R1* by protein–protein interactions in GBC.

Importantly, *MMP12* of Module 3 was validated as a candidate indicator of the extracellular matrix pathway that was the most significantly enriched in the functional network. Interestingly, *MMP12* may have an indirect effect on *PIK3R1* through *JUP* according to FI prediction (Figure 2A). *MMP12* is a member of the matrix metalloproteinase family, which is involved in the development and progression of malignancies (39, 40). *MMP12* mRNA expression was the most upregulated gene, with a 9.38-fold change in DEGs (Figure 2B), which is consistent with the protein expression trend (Figure 3F; *P* = 0.006). In contrast to *MMP12* as tumor suppressor, as reported by a previous study, our prognostic analysis showed that *MMP12* was protective against GBC (Figure 4C; *P* = 0.006). Furthermore, multivariate Cox analysis implied that *MMP12* is an independent protective factor for GBC (HR = 0.429; 95% CI 0.198, 0.930; *P* = 0.032). This controversial finding also is seen in other tumors; for instance, patients with hepatocellular or colon carcinomas whose tumors express *MMP12* mRNA have better survival than those whose tumors do not express *MMP12* and thus do not produce angiostatin (41, 42). In a squamous-cell carcinoma study, *MMP12* had a dual role in tumor progression (43). Meanwhile, we found that *MMP12* showed different trends among these common gastrointestinal cancers (Figure 6C). These discrepant results suggest that the role of *MMP12* in cancer progression differs between tumor or cell types. Moreover, a positive effect of *MMP12* on *PIK3R1* (*r* = 0.664; *P* < 0.0001; Figure 5D) was observed, suggesting *MMP12* has an indirect effect on *PIK3R1* through *JUP*.

Finally, the other candidate functional modules were screened, some of which contain genes that have already been investigated for their physiological function in carcinogenesis and tumorigenesis. Module 1 contains five DEGs (i.e., *LMOD1, MYH10, MYH11, MYL9*, and *SORBS1*) that were all downregulated in GBC. The enriched pathways of Module 1 are related mainly to muscle biology. *MYH10, MYH11*, and *MYL9* are members of the myosin family, a structural component of muscle. Recent research has found that myosins play important roles in cancer (44–46). *SORBS1* encodes sorbin and SH3 domain containing 1, the SH3 domains of which play a role in this protein's ability to bind other cytoplasmic molecules and contribute to cytoskeletal organization, cell adhesion and migration, signaling, and gene expression. Downregulation of *SORBS1* has been found in breast and prostate cancer (47, 48). Module 2, comprising *F13A1, FGA, FGB*, and *FGG*, is connected to Module 4, including *COL10A1* and *COL14A1*, by *ITGAX*. A hub node in two modules, *ITGAX* is also known as *CD11C* and encodes the integrin-alpha X-chain protein, which is one of the four members of the β2 leukocyte integrin family. *ITGAX* is involved in various immunologic functions, including cell adhesion, migration, and phagocytosis (49–51). Expression of *ITGAX* is evident in aggressive prostate cancer (52). *ITGAX* can interact with extracellular matrix molecules such as fibrinogen (53) and collagen (54). It links Modules 2 and 4, with Module 2 related to fibrinogen and Module 4 to collagen. Proteins *FGA, FGB*, and *FGG* are components of fibrinogen, and *F13A1* is a part of coagulation Factor XIII. Previous studies indicated that fibrinogen modulates angiogenic mechanisms to affect tumor growth and metastasis (55). Plasma fibrinogen overexpression was reported as an independent prognostic marker in GBC (56, 57). *COL10A1* encodes the alpha chain of type X collagen, a biomarker that is upregulated in a variety of tumors (58). The remaining genes either are the subject on only scant literature related to their involvement in cancer or showed results somewhat inconsistent with those of previous studies. Thus, experiments should be conducted to verify our findings. Taken together, these functional modules may comprise novel diagnostic markers for GBC.

Overall, *PIK3R1, LIFR*, and *MMP12* have never been reported in association with GBC. We first identified *LIFR, PIK3R1*, and *MMP12* as novel prognostic biomarkers in this cancer. Additionally, we built a highly reliable GBC-related FI network of dysregulated pathways to reveal a pool of novel functional module genes for further investigation in GBC development and progression, which might provide targets for therapy.

## Conclusions

In sum, our data demonstrated the effectiveness of network-based module analysis for biomarker discovery using gene expression data from GBC. Importantly, *LIFR, PIK3R1*, and *MMP12* have been revealed as novel prognostic signatures that might be targets. The findings of this study could accelerate biomarker discovery and therapeutic development in GBC.

## Data Availability

Publicly available datasets were analyzed in this study. This data can be found here: https://www.ncbi.nlm.nih.gov/geo/query/acc.cgi?acc=GSE62335.

## Ethics Statement

This study was carried out in accordance with the recommendations of the Ethical Committees of the National Engineering Center for Biochips at Shanghai and Taizhou People's Hospitals with written informed consent from all subjects. All subjects gave written informed consent in accordance with the Declaration of Helsinki. The protocol was approved by the Ethical Committees of the National Engineering Center for Biochips at Shanghai and Taizhou People's Hospitals. The use of human tissue samples and clinical data were approved by the Ethical Committees of the National Engineering Center for Biochips at Shanghai and Taizhou People's Hospitals.

## Author Contributions

XZ, MX, and ZC participated in data collection and analysis. XZ, MX, WY, and WC performed the laboratory experiments. XZ and ML conceived the study and wrote the paper. All authors approved the final manuscript.

### Conflict of Interest Statement

The authors declare that the research was conducted in the absence of any commercial or financial relationships that could be construed as a potential conflict of interest.

## Abbreviations

DEGs: differentially expressed genes
FI: functional interaction
GBC: gallbladder cancer
GO: gene ontology
MCL: markov cluster algorithm
PCC: pearson correlation coefficient.
